# Supplementary material for: Are carbon emissions trading and green financial instruments synergistic? -Comprehensive quantitative research based on content analysis
Source: PLoS One. 2024 Mar 7;19(3):e0298601. doi: 10.1371/journal.pone.0298601 (PMC10919601; doi:10.1371/journal.pone.0298601)
Supplement: S1 Data — (ZIP) [file pone.0298601.s001.zip › Data/地级市Z_F工作报告.zip!/║■─╧/╓Ω╓▐╩╨/└┤╫╘╣1⁄2╓┌║┼í╛┬φ┐╦╩2╛▌═°í┐_2019.docx]

各位代表：

现在，我代表市人民政府向大会报告工作，请予审议，并请各位政协委员提出意见。

一、2018年工作回顾

2018年是改革开放40周年。站在新的历史起点，我们在省委、省政府和市委的坚强领导下，坚持以习近平新时代中国特色社会主义思想为指导，认真落实高质量发展要求，以供给侧结构性改革为主线，着力打好三大攻坚战，深入开展产业项目建设年活动，统筹推进各项工作，经济运行总体平稳，高质量发展态势更加明显。预计全市地区生产总值增长7.8%，规模工业增加值增长7.4%，固定资产投资增长7%，一般公共预算收入同口径增长6.2%，社会消费品零售总额增长9.8%，城乡居民收入增长8.5%。税收占一般公共预算收入比重达到80.1%，同比提高12个百分点。

&mdash;&mdash;这一年，脱贫攻坚战取得决定性胜利。茶陵、炎陵两个国家片区贫困县成功脱贫摘帽，全市166个贫困村全部退出，比全国提前三年实现了整体脱贫和全面小康。

&mdash;&mdash;这一年，中国动力谷战略有力推进。&ldquo;3+5+2&rdquo;产业体系总产值达到2550亿元，增长16%，占规模工业比重72%，中国动力谷成为中国制造新名片。

&mdash;&mdash;这一年，老工业城市转型发展示范区建设取得标志性进展。随着株冶全面关停，标志着清水塘老工业区的工业企业全部关停退出，圆满实现&ldquo;三年大见成效&rdquo;的既定目标。

&mdash;&mdash;这一年，全面深化改革迈出坚实步伐。&ldquo;最多跑一次&rdquo;改革取得重大突破，九成以上事项实现&ldquo;最多跑一次&rdquo;，市民中心正式运行，实现&ldquo;中心之外无审批&rdquo;。土地节约集约利用工作获国务院通报表扬。

&mdash;&mdash;这一年，城市声誉和影响力不断提升。连续三年跻身全国百强城市，成功入选 &ldquo;改革开放40年来经济发展最成功的40个城市&rdquo; &ldquo;中国大陆最佳地级城市30强&rdquo;和&ldquo;产业竞争力百强城市&rdquo;。创建国家公共文化服务体系示范区验收成绩中部第一、全国第二，获批建设国家创新型城市。

过去一年，我们主要做了以下工作。

（一）以开展产业项目建设年活动为抓手，振兴实体经济。项目建设力度加大。大力实施&ldquo;五大行动&rdquo;，推进&ldquo;五个一批&rdquo;，投资70亿元的株洲信息港、投资50亿元的奇点汽车、投资40亿元的华录湖南数据湖产业园等280个重点项目顺利开工，竣工项目122个。入选国家智能制造项目10个、省&ldquo;五个100&rdquo;优秀项目5个，均居全省第二。完成集体土地征拆4.28万亩、国有土地房屋征收46.38万平方米、城市拆违141.8万平方米。新建标准厂房140万平方米。实施企业上市培育工程，推广&ldquo;投贷保补&rdquo;联动等投融资新模式，新增社会融资513亿元。产业结构不断优化。第三产业占比提高7.3个百分点，对GDP贡献率超过50%。炎帝陵创建国家5A景区进展顺利。制造业比重提高到92%。园区规模工业增加值占全市比重提高到76%，荷塘工业集中区、攸县工业集中区获批省级高新区，株洲高新区、醴陵经开区获评全省开放型经济优秀园区。创新引领作用加强。国家知识产权示范城市通过复核，城市创新竞争力跻身全国百强、全省第二。全市高新技术产业投资增长83.9%，全社会研发投入占GDP比重达到2.6%，新认定科技成果转化项目124个，引进科技创新人才270人，高新技术企业突破400家，高新技术产业增加值突破500亿元，占规模工业增加值比重超过60%。全国首列商用磁浮2.0版列车、全球首条智能轨道示范线、生物医药多肽库等一批重大创新成果在株洲面世，&ldquo;复兴号&rdquo;高铁、AG600大型水陆两栖飞机、港珠澳大桥等大国重器闪耀着株洲元素。

（二）以打好三大攻坚战为重点，补齐发展短板。风险防控有力有效。制定实施防范化解政府债务&ldquo;30条&rdquo;，平台公司市场化转型整合完成年度任务，&ldquo;停缓调撤&rdquo;压减投资757亿元，政府债务风险等级下降。重拳整治金融市场乱象，&ldquo;处非&rdquo;工作连续四年全省第一。市级金融安全区创建工作全面完成，醴陵、炎陵、攸县获得省级金融安全区授牌。脱贫攻坚决战决胜。大力实施&ldquo;七个一批&rdquo;和&ldquo;六大工程&rdquo;，启动巩固脱贫成果三年行动，开展扶贫领域作风问题专项治理，新一轮驻村帮扶实现全覆盖。全年减少贫困人口2.58万人，贫困发生率由1.3%下降到0.43%。社会扶贫模式在世界互联网大会上作推介。环境污染严防严治。大力推进&ldquo;四战两行动&rdquo;和&ldquo;夏季攻势&rdquo;。全力开展&ldquo;五控&rdquo;等专项整治行动，市区空气质量优良天数达到288天，较上年增加16天，居长株潭三市之首。湘江保护和治理第二个&ldquo;三年行动计划&rdquo;圆满收官，河长制工作全面展开，黑臭水体治理年度任务全面完成，全市水体水质稳步改善，成功创建全国水生态文明城市。实施&ldquo;绿盾2018&rdquo;和国土绿化行动，新造林17万亩，绿心地区工业企业全面退出，水土流失治理、退耕还林还湿取得积极成效。完成种植结构调整29.2万亩，修复治理重金属污染耕地4万亩，休耕16.3万亩。突出抓好中央、省环保督察问题整改和中央环保督察&ldquo;回头看&rdquo;问题办理。&ldquo;清废行动2018&rdquo;专项督查交办问题全部整改到位。

（三）以深化改革开放为动力，激发发展活力。重点领域改革取得新突破。&ldquo;三去一降一补&rdquo;成效明显，清水塘老工业区153家企业全部关停，钢铁、烟花鞭炮过剩产能全部退出，转移转型步伐加快。房价涨势得到控制，企业负债率持续下降。&ldquo;放管服&rdquo;改革深入推进，工业项目实现&ldquo;拿地即开工&rdquo;，新建楼盘&ldquo;交房即交证&rdquo;。创新审批管理经验在全省推介。&ldquo;证照联办&rdquo;改革全面启动，&ldquo;减证便民&rdquo;专项行动精简事项90%，涉企证照实现&ldquo;四十九证合一&rdquo;，企业开办时间压缩至3个工作日以内，新增市场主体4.1万户。在全国率先推行财政投资评审负面清单管理，在全省率先制订政府采购负面清单、推行经营性资产集中统管和财政性资金竞争存放管理。中央省属企业&ldquo;三供一业&rdquo;分离移交走在全省前列。国土空间规划成为全省唯一试点市。农村土地确权登记颁证通过验收。获批全省唯一的&ldquo;全国农村集体产权制度改革&rdquo;整市试点。国有农场社会职能改革经验全省推广。城市管理执法体制改革绩效考核排名全国第六、全省第一。全国城市管理片区工作会议在我市召开，城市管理工作经验再次在全国推介。开放发展水平不断提升。大力推进&ldquo;五大开放行动&rdquo;，铜塘湾保税物流中心封关运营，湘欧快线株洲始发首列班车即将开通，铁海联运无水港等项目加快推进。成功举办湖南国际旅游节暨瓷博会、国际茶文化研讨会、服博会等品牌节会，积极参与&ldquo;长洽周&rdquo;&ldquo;进博会&rdquo;等重大经贸活动。组建三个招商联络处驻外招商，签约引进项目311个，其中 &ldquo;三类500强&rdquo;企业项目49个。全市进出口总额增长30.8%，其中与&ldquo;一带一路&rdquo;沿线国家和地区贸易额增长48%。

（四）以推动国家新型城镇化综合试点为契机，改善城乡面貌。城镇品质不断提升。主动融入长株潭一体化，加快推进牵头的4项工作，&ldquo;交通一卡通&rdquo;在长株潭全面推行，区域协同发展迈出新步伐。纵深推进&ldquo;五大行动&rdquo;，神农大道A段、龙洲路、枫溪大道拓宽改造等一批项目顺利竣工，莲株高速、湘江大道一期建成通车，东城大道即将全线贯通。绿色交通城市完成部省验收，市区新能源公交车实现全覆盖。顺利通过国家园林城市第三次复查。航电枢纽二线船闸建成通航，湘江株洲段通航标准由1000吨提升至2000吨。完成老旧小区专项整治项目13个2549套，改造棚户区住房2.29万套，老旧住宅加装电梯试点顺利推进。改造小街小巷和支路55条，新增公共停车位2050个。大力推动&ldquo;厕所革命&rdquo;，100座&ldquo;24小时不打烊&rdquo;的&ldquo;建宁驿站&rdquo;建成开放。渌口区正式挂牌，&ldquo;株醴融城&rdquo;步伐加快，城市发展空间大幅度拓展。特色镇建设持续推进，20个特色镇实现燃气全覆盖，一批乡镇污水处理设施建成运营，县以上城镇生活垃圾无害化处理率达到100%。出台市政公用设施移交管理办法，符合条件的市政公用设施全面移交到位。启动新一轮城市总体规划修编工作。率先在全省颁布实施农村村庄规划建设管理条例，实现村镇规划全覆盖。乡村振兴战略开局良好。扎实推进现代农业特色产业集聚区建设，新增省级现代农业特色产业示范园4个，新建农业标准化示范基地20个。龙头企业、农民合作社、家庭农场等新型农业经营主体不断壮大。农村产业融合步伐加快，农产品加工总产值达602亿元。农村人居环境整治全面启动，完成250个村的农村环境综合整治，积极创建&ldquo;四好农村路&rdquo;，完成自然村通水泥（沥青）路771公里，提质改造农村公路288公里。除险加固水库104座，解决和巩固23.8万农村人口饮水安全问题。成功创建省级美丽乡村示范村4个、市级示范村25个。乡风文明焕发新气象，建成基层综合文化服务中心920个，6人入选&ldquo;中国好人榜&rdquo;。农村治理体系更加完善，108个乡镇（街道）、1379个村（社区）建成网格化服务管理平台，1065个村建成驻村警务室并配备驻村辅警。

（五）以实施民生100工程为载体，增进民生福祉。社会保障不断加强。民生100工程逐一落实，省重点民生实事全面完成。新增城镇就业和农村劳动力转移就业超额完成年度任务，城镇登记失业率降至2.23%。企业退休人员基本养老金实现&ldquo;14连调&rdquo;。居家和社区养老服务改革国家试点系列做法在全省推广。基本医保、大病保险和民政医疗救助实现&ldquo;一站式&rdquo;结算。在全省率先对80岁以上老人发放高龄津贴。市区城乡低保标准从470元/月提高至520元/月。发放城镇住房保障家庭租赁补贴6381户，农村危旧土坯房改造全面完成。社会事业全面进步。学前三年入园率达94.1%。超大班额全部&ldquo;清零&rdquo;，义务教育巩固率达98.7%。农村学校&ldquo;全面改薄&rdquo;任务圆满完成。市属汽车工程职院、工业学校、幼师学校项目建设基本完成，长郡株洲云龙实验学校、南方中学新校区开工建设。湖南工业大学成功升为一本院校。市中心医院田心院区提质改造基本完成。分级诊疗制度全面推开，组建医疗联合体27个。荣获全省人口和计生工作优秀单位。成功蝉联全国无偿献血先进城市。第二轮市志修编全面完成。市戏剧传承中心顺利搬迁，市文化馆正式对外开放。株洲河西杜甫草堂修复工程启动。株洲国家文化消费试点平台建成。成功承办第六届湖南艺术节、首届中国&middot;株洲国际赛车文化节。《英&middot;雄》获评田汉大奖，作为湖南唯一剧目入选第十二届中国艺术节。在第十三届省运会上获得第三名的好成绩。社会治理力度加大。深入推进扫黑除恶专项斗争，铲除了一批涉黑组织和涉恶团伙，全市刑事发案率和治安发案率大幅下降。深入开展&ldquo;三无创建&rdquo;&ldquo;四大攻坚&rdquo;&ldquo;信访法治化建设&rdquo;活动，打好安保维稳&ldquo;百日会战&rdquo;，进京非正常上访大幅下降，特护期进京非访实现零登记。健全&ldquo;四巡四防&rdquo;防控机制，推进&ldquo;雪亮工程&rdquo;，启动摩托车、电动车&ldquo;物联网&rdquo;防控系统建设。深化&ldquo;落实企业主体责任年&rdquo;，荣获全省安全生产工作优秀单位。启动国家食品安全示范城市创建工作，全年食品药品安全&ldquo;零事故&rdquo;。成功将非洲猪瘟疫情阻击在株洲市域之外。密切军政军民团结，国防动员和后备力量建设持续加强，优抚安置等双拥共建工作取得新成果。

（六）以提高依法行政水平为目标，加强自身建设。认真落实全面从严治党要求，不断巩固&ldquo;两学一做&rdquo;学习教育常态化制度化成果，严格落实中央八项规定精神，坚决纠正&ldquo;四风&rdquo;问题，全面落实巡视反馈问题意见整改，廉政建设和反腐败斗争不断加强。纵深推进&ldquo;马上就办、真抓实干&rdquo;活动，剑指不作为、慢作为、乱作为。全力推进覆盖市县乡村四级的&ldquo;互联网+政务服务&rdquo;平台和基层治理综合管理服务体系建设，建成全市统一的基础大数据库，入库数据资源的数量和质量居全省前列。新建惠民综合服务中心（社）240个。12345市长热线扩容升级，全年受理群众咨询投诉建议11万件，办结率、满意率稳步提升，在第二届全国12345政府服务热线高质量研讨会上荣获&ldquo;骏马奖&rdquo;。配合市人大常委会制定出台工业遗产保护法规。认真执行市人大及其常委会的决议和决定，自觉接受市人大及其常委会的法律监督和工作监督，主动接受市政协的民主监督和社会舆论监督。定期向市人大报告工作，向市政协通报情况。积极处理各类审议意见，全年办理人大代表建议314件、政协提案253件，见面率、办结率、满意率均为100%。认真吸纳各民主党派和工商联调研成果。加强法治政府建设，首次组织新任命国家工作人员参加宪法宣誓。全面推行法律顾问制度，严格规范政府行为，依法行政水平明显提高。

与此同时，统计、审计、质监、海关、外事侨务、人防、国安、移民、供销、广电、新闻出版、档案、地方志、气象、农机、水文、民族宗教、住房公积金、对口支援、妇女儿童、残联、文联、红十字、贸促、侨联、对台等工作都有新进步。

各位代表！看似寻常最奇崛，成如容易却艰辛。我们深深地体会到，这些成绩的取得，党的领导是根本保证。旗帜指引方向，火炬照亮前程，我们高举习近平新时代中国特色社会主义思想伟大旗帜，在省委、省政府和市委的坚强领导下，汇聚起奋进新时代开启新征程的磅礴伟力。面对经济下行压力、政府债务化解、污染防治攻坚等多重挑战，全市广大干部群众不畏艰险，保持定力，担当作为，攻坚克难，奋力谱写了新时代的株洲篇章。这些成绩的取得，离不开市人大、市政协的监督与支持，离不开社会各界的关心和帮助，更离不开全市人民的团结奋斗。上下同心，其利断金。只要408万株洲人民心往一处想，劲往一处使，就没有什么困难不能战胜，没有什么奇迹不能创造！在此，我代表市人民政府，向全市广大干部群众，向人大代表、政协委员，向各民主党派、工商联、无党派人士和人民团体，向驻株部队官兵以及中央、省驻株单位，向离退休老同志，向所有关心支持株洲发展的各界朋友，表示衷心的感谢和崇高的敬意！

在总结成绩的同时，我们也清醒看到问题和不足：一是经济下行压力加大，经济增速放缓，部分企业经营困难，传统动能大幅削减，发展新动能仍然不足。二是风险隐患化解任务还比较重，财政收支矛盾突出，平台公司转型和环境污染治理任务艰巨，安全生产、信访维稳等领域压力较大。三是民生改善与群众期盼还有差距，就学、就医、社保、住房、养老等方面仍需加大工作力度，交通拥堵、停车难等问题仍需加强整治。四是部分干部的作风与高质量发展要求还不相适应，政府治理体系和治理能力现代化水平有待提升。对此，我们要高度重视，认真解决。

二、2019年形势和任务

今年是新中国成立70周年，是着力推动高质量发展、加快建成&ldquo;一谷三区&rdquo;，加快实现基本现代化的重要一年。当前宏观形势错综复杂，外部环境更加严峻，不确定性更加凸显。同时，我市结构调整阵痛期、新旧动能转换期、债务偿还高峰期、污染防治攻坚期叠加，矛盾和问题更加突出，必须做好打硬仗的准备。但从长远和趋势看，我们仍处于并将长期处于重要战略机遇期，经济发展长期向好的基本面没有变，经济持续增长的良好支撑基础和条件没有变，结构调整优化的前进方向没有变。中央继续实施积极的财政政策、稳健的货币政策，制定实施促进中部崛起规划。长株潭城市群效应和我市众多&ldquo;国字号&rdquo;政策效应日益彰显，中国动力谷支撑引领作用明显增强，&ldquo;最多跑一次&rdquo;等关键改革释放新动能。特别是改革开放40年来，勤劳智慧的株洲人民以奋勇争先、一往无前的&ldquo;火车头&rdquo;精神，勇立潮头、破浪前行，书写了&ldquo;神农福地、动力之都&rdquo;的壮丽史诗，让我们更有信心、更有力量，抢抓新机遇，创造新辉煌！

今年政府工作的总体要求是：以习近平新时代中国特色社会主义思想为指导，认真落实党中央、国务院和省委、省政府，以及市委的决策部署，坚持稳中求进、进中争先工作总基调，坚持新发展理念，坚持推动高质量发展，坚持以供给侧结构性改革为主线，坚持深化市场化改革、扩大高水平开放，坚持创新引领开放崛起，继续开展产业项目建设年活动，继续打好三大攻坚战，继续实施民生100工程，着力开展温暖企业行动，着力稳就业、稳金融、稳外贸、稳外资、稳投资、稳预期，坚定信心、保持定力、迎难而上、担当作为，加快建成&ldquo;一谷三区&rdquo;，加快实现基本现代化，以优异成绩庆祝中华人民共和国成立70周年。

围绕推动高质量发展，今年主要预期目标是：地区生产总值增长8%左右，规模工业增加值增长7.5%，固定资产投资增长10%，一般公共预算收入增长7%，社会消费品零售总额增长10%，居民收入增速高于经济增速，金融财政风险有效防控，生态环境进一步改善，完成省下达的节能减排等约束性指标。

为实现上述目标，要重点把握以下几点：

&mdash;&mdash;必须坚定信心决心。株洲发展正处于爬坡过坎、不进则退的关键时期。信心决不可动摇，干劲决不可松懈。我们务必紧扣重要战略机遇新内涵，坚定必胜信心、争胜决心，善于化危为机，转危为安，变压力为动力，在新起点上把各项工作提高到新水平。

&mdash;&mdash;必须坚持高质高效。坚持质量第一、效益优先、安全至上、创新为要，聚焦加快建成&ldquo;一谷三区&rdquo;总任务，努力在促进制造业高质量发展、提高企业效益上取得新进展，在深化市场化改革、扩大高水平开放上实现新突破，在污染治理、绿色发展上创造新业绩，在改善财税质量、提高居民收入上取得新成效。

&mdash;&mdash;必须坚决为民惠民。始终坚持全心全意为人民服务的根本宗旨，践行以人民为中心的发展思想。倾听人民心声，汲取人民智慧。多办利民实事，多做惠民好事，多解民生难事。不断提高教育、养老、医疗、文化等公共服务供给质量，不断提升人民群众的获得感、幸福感、安全感。

&mdash;&mdash;必须坚守底线红线。自觉把底线思维和红线意识贯穿于各项工作中，处理好防风险与稳增长的关系，既要持续打好三大攻坚战，坚决守住金融、生态、安全生产、信访维稳等领域的风险底线，也要算好发展账、民生账、债务账，统筹做好各项政策、要素的协同配合，做到肩上有责，心中有数，手里有招。

各位代表！要完成2019年各项目标和任务，我们既要有&ldquo;乱云飞渡仍从容&rdquo;的战略定力，又要有&ldquo;不到长城非好汉&rdquo;的进取精神。走进伟大的新时代，让我们撸起袖子，挥洒汗水，朝着&ldquo;无限风光在险峰&rdquo;的理想之巅奋力攀登，绘就株洲高质量发展的壮美画卷！

三、2019年重点工作

围绕以上目标和要求，重点抓好以下工作：

（一）致力推动产业高质量发展

加快发展先进制造业。大力推动制造强市&ldquo;1093&rdquo;行动，加快推进制造业提质升级。做强产业集群。坚持以中国动力谷建设为统揽，着眼强链补链延链，聚焦15个优势产业链，完成轨道交通、汽车、航空、电子信息等产业规划的修编审定。继续推进工业&ldquo;十百千&rdquo;工程，引进配套企业100家以上，新增规模工业企业100家以上，不断提升&ldquo;3+5+2&rdquo;产业体系竞争力，全力将轨道交通产业打造成世界级产业集群，将航空、新能源汽车、电子信息、新材料等产业打造成国家级产业集群。加快创建国家安全产业示范区，建设中国安全谷。提升智能制造水平。推进云计算、大数据、人工智能等现代信息技术和制造业的深度融合，加快陶瓷、服饰、烟花鞭炮等传统产业的改造升级，壮大生物医药、新能源等战略性新兴产业，创建一批智能化示范企业和智能制造示范车间，争创制造业高质量发展示范区。做优品质品牌。大力实施质量强市战略，开展全国质量强市示范城市创建活动。推进轨道交通、通用航空、硬质合金、陶瓷、服饰产业集群区域品牌试点建设，大力弘扬企业家精神和工匠精神，培育一批名牌产品和品牌企业。强化项目支撑。按照&ldquo;签约项目抓落地、开工项目抓保障、在建项目抓进度&rdquo;的思路，不断完善工作机制，以实物量带动投资量、形成新产能，确保全年产业投资超过1000亿元。加快推进IGBT二期、华录（湖南）数据湖、火炬特种高纯功能靶材、高端PI薄膜、&ldquo;两机&rdquo;重大专项、奇点汽车、汉德车桥、株冶锌基材料、中小型航机燃机等项目建设。强化资金、土地等要素保障，新增社会融资400亿元以上，完成集体土地征拆3万亩、国有土地房屋征收30万平方米。

大力推进科技创新。以创建国家创新型城市为契机，推动国家自主创新示范区政策向全市范围延伸，实施全社会研发经费投入三年行动计划，力争全社会研发投入占GDP比重达到2.7%。以企业为主体推进自主创新，培育创新领军企业和&ldquo;专精特新&rdquo;科技型中小企业，新增高新技术企业100家，实现高新技术产业增加值600亿元。打造株洲&ldquo;双创&rdquo;升级版，做优&ldquo;在线孵化器&rdquo;&ldquo;创新研究院&rdquo;&ldquo;创新创业投资基金&rdquo;等公共服务产品，新增孵化器面积20万平方米，科技成果转化100项。突出抓好轨道交通装备制造业创新中心、功率半导体制造业创新中心等创新平台建设，争取新能源汽车研究院、新能源汽车国检中心等研发平台落户株洲。全面落实人才新政&ldquo;30条&rdquo;，组建&ldquo;千里马&rdquo;&ldquo;梧桐树&rdquo;基金，引进科技创新人才200名。以创建国家军民融合创新示范区为抓手，开展知识产权军民融合试点，建设一批军民融合重点项目，做强航空等军民融合产业示范基地。

发展壮大园区经济。树立园区即&ldquo;特区&rdquo;的理念，全力推进园区升级三年行动计划，着力打造创新创业园区&ldquo;135工程&rdquo;升级版。完善园区功能配套。推进&ldquo;五张网&rdquo;向园区延伸，健全学校、医院等公共服务配套设施，推进园区&ldquo;二次开发&rdquo;，新建标准厂房120万平方米，提升园区承载能力。推进园区晋位升级。压实园区招商引资责任，力争园区新引进工业项目100个，新开工工业项目100个，支持醴陵经开区、株洲经开区创建国家级经开区。加快园区体制创新。推广园区管理新模式，推动园区平台公司市场化转型和投融资体制改革，进一步理顺园区体制机制。探索建立园区高质量发展考核指标体系，在河西示范园、田心高科园率先启动亩产效益评价，组织开展园区季度考评，及时兑现考核奖励。

支持发展民营经济。认真落实习近平总书记在民营企业座谈会上的重要讲话精神，坚持&ldquo;两个毫不动摇&rdquo;，积极开展温暖企业行动，让民营经济创新源泉充分涌流、创造活力充分迸发。更大力度减税降费，开展减税降费专项督查，推动民营经济政策全面落地。持续优化市场准入，建立健全民间资本投资重点领域项目库，对民间投资进入资源开发、交通、市政等领域，除另有规定外一律取消最低注册资本、股本结构等限制。不断创新融资服务，加强财政、信贷、产业政策联动，全面落实金融扶持小微企业发展政策和风险补偿制度，建立健全市县两级融资担保体系，鼓励银行扩大对小微企业的信贷投放力度，着力解决企业融资难融资贵问题。鼓励帮扶企业发展，大力实施&ldquo;个转企、小升规、规改股、股上市&rdquo;行动，新增&ldquo;个转企&rdquo;500家、&ldquo;规改股&rdquo;25家、&ldquo;四上&rdquo;企业500家。着力构建&ldquo;亲&rdquo;&ldquo;清&rdquo;新型政商关系，切实保护民营企业家人身和财产安全，任何政府部门、国有企业，都不得违约拖欠中小企业款项，作出的各项承诺必须坚决依法兑现！

加快促进服务业发展。推进先进制造业与现代服务业融合发展，补齐科创、信息、商务、人力资源等生产性服务业短板，做强金融、电商、工业设计等生产性服务业，培优商贸、文化、健康、养老、体育等生活性服务业。突出抓好服务业集聚区提升工程，推动服务业发展提速、比重提高、层次提升。积极发展现代物流，抓好县乡村三级物流配送体系建设三年行动计划，加快中车物流基地、株洲农产品物流中心及县域物流园建设，完善农村物流网布局，打造长株潭区域性物流中心。着力发展全域旅游，挖掘工业游、生态游等旅游业态潜力，开发特色商品，做活旅游地接，发展中国动力谷工业旅游，加快炎陵、醴陵创建全域旅游示范县步伐，打造湘东旅游带，努力建设&ldquo;国内知名旅游目的地&rdquo;。大力发展会展经济，举办陶瓷、服饰、烟花、茶叶等特色展会，以及食祖文化节等系列节会活动。继续推进农贸市场改造升级。完善市场监管体系，切实保护消费者合法权益。

（二）致力推进城乡统筹发展

主动融入长株潭一体化。全面落实长株潭城市群一体化发展首届联席会议精神，推动14个方面和20项重点任务落地见效，着力推进规划、交通、产业、公共服务、环境治理&ldquo;五个一体化&rdquo;取得新进展。积极参与长株潭城市群区域规划、生态绿心地区总体规划等修编工作，开展&ldquo;长株新城&rdquo;可行性研究。推进洞株公路株洲段快速化改造，加快长株攸公路等项目建设，推进渝长厦支线、长沙南到株洲磁浮、长沙地铁3号线南延至株洲等项目前期，优化交界地区公共交通网络，打通城际间&ldquo;断头路&rdquo;，打造&ldquo;半小时交通圈&rdquo;。加强株洲经开区与长沙南部新区联动发展，株洲高新区与湘潭天易示范区协调发展，在产业融合错位发展中争取先机。推动图书馆、文化馆、博物馆等公共服务资源共享。推进&ldquo;绿心&rdquo;、湘江流域等区域生态环境的联防联治联控，共护长株潭碧水蓝天青山。

持续提升城镇承载能力。推动规划布局升级。坚持推进&ldquo;多规合一&rdquo;，全面启动国土空间规划编制，推进城市总体规划、县城总体规划修订和村镇规划修编，形成产业、国土、环保等规划成果&ldquo;一张图&rdquo;。做大做强中心城区核心增长极，加快南洲新区及武广、新马、枫溪、水竹湖、清水塘、云龙绿地文化旅游城、华夏幸福等新城开发，加快渌口区在基础设施、产业发展、城市管理等方面的融城步伐，推进株醴都市圈建设，扩大城市人口规模，拓展城市发展空间。着力打造醴炎县域经济走廊，加快攸县撤县设市进程，强化县城和特色城镇、中心集镇建设，打造瓷谷小镇、航空小镇、轨道小镇等一批特色产业小镇。推动功能品质升级。继续实施&ldquo;五大行动&rdquo;，推进城市&ldquo;双修&rdquo;，加快&ldquo;城市更新&rdquo;。开工建设醴娄高速等项目，力争茶界高速开工、长株潭东外环线高速纳入省高速公路网，实现城区高速互通10公里半径全覆盖。全力推进株洲火车站改扩建工程，打造城市综合交通枢纽。推进渌水、洣水保护与开发。启动创建国家生态园林城市三年行动，加快&ldquo;两岸一线&rdquo;&ldquo;十园十湖&rdquo;建设，基本建成雪峰岭公园、凤凰山公园、新苗公园，加快轨道交通城中央公园、博古山公园、明照湖蓄洪生态公园、神农生态带、云峰森林植物园建设。启动清水塘工业遗址公园建设，加快河东风光带文化长廊工程和河西杜甫草堂修复工程建设，打造白石人家、建宁记忆、枫溪渔港等株洲历史文化景观。加快老旧小区改造，推进田心、董家塅、&ldquo;430&rdquo;等片区配套设施改造更新，推动有条件的老旧住宅加装电梯。大力实施城区&ldquo;厕所革命&rdquo;，全面完成新建、改造、开放厕所各200座。推进海绵城市建设，补齐雨污分流、排水防涝等基础设施短板。启动电网建设三年行动计划，再造一个株洲电网。推动管理服务升级。严格执行《株洲市城市综合管理条例》，提升城市管理科学化、精细化、智能化、法治化、社会化水平。升级改造数字城管平台，强化园林绿化建设质量监管，加强城市管理考评。深入开展户外广告招牌、违法违规建设等专项整治行动。拓展文明创建的广度和深度，擦亮全国文明城市&ldquo;金字招牌&rdquo;。

大力实施乡村振兴战略。落实乡村振兴战略规划，推进&ldquo;五大振兴&rdquo;，加速农业农村现代化进程。壮大农业产业。推进现代农业特色产业集聚区建设&ldquo;六大行动&rdquo;，力争创建省级现代农业特色产业集聚区1个、省级优质农副产品供应示范基地2个。加快调整农业产业结构，实施农作物种植结构调整28万亩。促进农产品加工业转型升级，实现农产品加工业总产值增长20%以上。实施农业品牌提升行动，做大白鹅、红茶、大豆、油茶、蔬菜、花卉、苗木等&ldquo;一县一特&rdquo;产业，做响株洲红茶、醴陵油茶、炎陵黄桃等区域公用品牌。加强农产品质量安全监管体系建设，推进60家农业标准化示范基地提质升级，新增&ldquo;三品一标&rdquo;认证农产品40个。加大新型主体培育力度，继续实施&ldquo;十企百社千户&rdquo;现代农业发展工程，新增市级以上龙头企业20家、市级示范农民合作社50个、市级示范家庭农场30个。改善人居环境。推进&ldquo;一廊十镇百村&rdquo;示范工程建设，建成省级示范村4个、市级示范村25个。大力实施农村人居环境整治三年行动，突出抓好&ldquo;五大工程&rdquo;，打造人居环境整治株洲特色和示范样本。推进农村饮水安全工程，完成7万农村人口饮水安全巩固提升任务。推进60座小型病险水库除险加固。加快&ldquo;四好农村路&rdquo;建设，实现农村地区基本公共客运服务全覆盖。抓好农村路边、水边、房边绿化，实现省道、县道、乡村道路&ldquo;应绿尽绿&rdquo;。大力推进农村&ldquo;厕所革命&rdquo;。深入实施&ldquo;光纤入村&rdquo;&ldquo;数字乡村&rdquo;&ldquo;气化株洲&rdquo;工程，让农村用气安全便捷、电视信号稳定、网络信号全覆盖。严格落实《株洲市农村村庄规划建设管理条例》，实现乡村建设规划和实用性村庄规划全覆盖。创新乡村治理。健全自治、法治、德治相结合的乡村治理体系，打造100个善治示范村。健全&ldquo;四议两公开&rdquo;等民主制度，实现政府治理和居民自治良性互动。加强农村文化建设，健全乡村公共文化服务体系。加大移风易俗力度，开展&ldquo;议家风、定家规、晒家训&rdquo;等活动，培育良好家风、文明乡风、淳朴民风。

（三）致力深化改革开放

深化以供给侧结构性改革为主线的各项改革。遵循&ldquo;巩固、增强、提升、畅通&rdquo;的方针，完成&ldquo;三去一降一补&rdquo;任务。深化&ldquo;放管服&rdquo;改革，加快推进&ldquo;互联网+政务服务&rdquo;，全面推行&ldquo;马上办、网上办、就近办、一次办&rdquo;。加快建设&ldquo;1+4&rdquo;综合服务平台，完善市民中心配套服务，建设县市区市民中心，实现&ldquo;最多跑一次&rdquo;改革市域全覆盖。全面完成政府机构改革任务。深化财税体制改革，加强预算绩效管理，进一步理顺城区事权和支出责任。推进国企国资改革，加快混合所有制改革，推行企业投融资担保负面清单管理。推进要素市场化改革，加快公共资源交易体制、信用体制改革。抓好产业链融资和无还本续贷工作，组建政策性融资担保公司，完善&ldquo;投贷保补&rdquo;联动机制。推进农村承包地&ldquo;三权分置&rdquo;，扎实开展农村集体产权制度改革整市试点。推进生态文明体制改革，在党政机关、学校推广生活垃圾分类。开展生态环境第三方评估改革试点。健全生态环境损害赔偿制度，建立完善环境损害赔偿两库一清单。继续做好第四次全国经济普查工作。推进第三次国土调查工作。大力推进综合执法、供销合作社等改革。

提升对外开放水平。深度对接&ldquo;一带一路&rdquo;、长江经济带、粤港澳大湾区和长株潭城市群等国省战略，深入实施&ldquo;五大开放行动&rdquo;，加快建设&ldquo;一带一部&rdquo;开放发展先行区。帮助企业开拓新兴国际市场，组织企业参加德国汉诺威工业博览会等会展活动。支持轨道交通、通用航空、新能源汽车、硬质合金、陶瓷、烟花等优势企业和优质产品出海，开展跨国并购，带动装备、产品、技术、服务等&ldquo;走出去&rdquo;。实施外贸企业培育计划，做大做强外贸综合体。落实国省鼓励进口各项政策，鼓励对核心零部件、先进设备、关键原材料的引进消化吸收再创新，力争进出口总额增长20%。优化&ldquo;平台+口岸&rdquo;布局，启动清水塘口岸经济园建设，加快跨境电商物流园、进出口机床及产品展示展销中心等项目建设，推动沪九直通车在株开设乘客进出口岸。继续加大招商引资力度，充分发挥驻外招商联络处作用，借力首届中非经贸博览会、中国国际轨道交通博览会、&ldquo;港洽周&rdquo;等重要经贸活动，力争全年引进项目200个，其中&ldquo;500强&rdquo;产业项目20个，过50亿元项目20个，建设湘商产业园2个。实际利用外资增长10%，内联引资增长15%。

（四）致力打好三大攻坚战

防控金融风险。坚持&ldquo;控增量、去存量、守底线&rdquo;原则，全面落实债务化解方案，采取年度财政预算安排一批、协商金融机构展期缓解一批、组建债务平滑基金缓释一批等措施，确保隐性债务不新增、借新还息不发生、&ldquo;三保&rdquo;支出不断链，实现债务总量、综合债务率和预警等级稳步下降。全面完成国有投资公司市场化转型整合，全面排查企业债券风险，降低国有企业杠杆率。牢固树立过&ldquo;紧日子&rdquo;的思想，压缩一般性支出，提高资金使用效率。深入开展非法金融活动风险专项整治，严密防控非法集资、民间融资、互联网金融等领域风险，坚决守住不发生系统性区域性金融风险的底线。

加强生态建设。持续推进&ldquo;四战两行动&rdquo;，让好山好水好风光遍布株洲大地。启动湘江保护和治理&ldquo;第三个三年行动计划&rdquo;，压实河长责任，推进&ldquo;一江两水&rdquo;等流域污染治理，抓好河湖&ldquo;清四乱&rdquo;工作和河湖管理范围划定工作。加快建宁港、白石港黑臭水体综合整治，完成荷叶塘干渠（下游）、人民北路至白石港排水渠等9处黑臭水体整治，建成王仙镇等22个乡镇污水处理厂。开展餐饮油烟整治，建成PM2.5、PM10监控小微站100个，推进市区二期、醴陵市、茶陵县生活垃圾焚烧发电厂建设，加快县市区渣土和建筑垃圾消纳场所和设施建设。启动实施铜塘湾、清水湖等片区污染土壤修复治理，完成重金属污染耕地修复治理4万亩、休耕12.7万亩。大力推进全域增绿行动，确保森林蓄积量和森林覆盖率稳中有升。全力抓好中央、省环保督察反馈问题以及第三方评估机构发现问题的整改。

巩固脱贫成果。深入开展巩固脱贫三年行动，认真落实&ldquo;四摘四不摘&rdquo;和&ldquo;五个全面&rdquo;的要求，进一步提升脱贫质量，巩固脱贫成果，确保全面小康路上&ldquo;一个不掉队&rdquo;。大力开展产业扶贫，加大资金投入，鼓励农业龙头企业、新型农业经营主体与贫困户建立利益联结机制，确保贫困人口参与产业扶贫覆盖面达到80%。大力提升保障水平，严格落实贫困对象危房改造、教育扶贫、健康扶贫、兜底保障等扶贫政策，确保未脱贫对象达到脱贫标准，已脱贫对象实现稳定脱贫。大力开展社会扶贫，充分发挥&ldquo;一张网、一个联盟、一支基金&rdquo;的作用，凝聚社会合力，对接贫困群众需求，助力扶贫产业发展。增强贫困群众内生动力，开展&ldquo;志智双扶，星级评比&rdquo;，营造良好脱贫氛围。大力转变干部作风，抓好扶贫领域作风问题专项治理，奋力夺取脱贫攻坚新胜利。

（五）致力保障和改善民生

提升创业就业质量。实施就业优先战略和更加积极的就业政策，确保全年新增城镇就业5万人、农村劳动力转移就业1.2万人，保持&ldquo;零就业家庭&rdquo;动态援助100%。实施高校毕业生就业创业促进计划和基层成长计划，鼓励高校、中专、技校学生到基层和企业工作。深入实施农民增收三年行动计划，引导支持返乡农民创业就业。支持发展就业容量大、市场前景好的劳动密集型产业，统筹做好关停搬迁企业职工、退役军人等就业安置工作。健全&ldquo;治欠保支&rdquo;长效机制，全面治理拖欠农民工工资问题，让劳动者更加体面生活，更受社会尊敬！

强化民生兜底保障。继续实施城镇棚户区改造，实现农村四类重点对象危房改造清零。全面实施全民参保计划，新增企业养老保险参保3万人，推进工伤保险市级统筹、农民工参保等工作。健全低保标准增长机制，确保农村低保标准不低于国家扶贫线，月人均补差提高到185元以上。完善落实社会救助、临时救助、特困人员救助等制度，资助家庭经济困难学生6万人次，帮扶救助困难职工1万人次，切实关心农村留守儿童和留守老人，免费提供残疾人辅助器具1100件，让每一位身处困境的人都能得到关爱和温暖！

抓好交通拥堵治理。紧扣&ldquo;出行难&rdquo;&ldquo;停车难&rdquo;等问题，努力改善出行条件。协调推动醴茶铁路提质改造。打通黄山西路、航空大道等&ldquo;断头路&rdquo;，疏通红港路与人民路交叉口、株醴路与南环路交叉口等交通堵点，加快推进新东路、响石广场等项目建设，新建改造小街小巷30条以上。规范停车管理，新建公共停车场4个，新增停车位1500个。完成城区巡游出租车改革，新增和优化公交线路4条，建成&ldquo;公交都市&rdquo;。完成公共自行车系统更新改造3000台（套）。完成自然村通水泥（沥青）路1100公里、窄路加宽100公里、安防工程400公里、危桥改造30座、新建农村客运招呼站50个。

办好公平优质教育。聚焦义务教育&ldquo;城市挤、农村弱&rdquo;的问题，改善农村办学条件，合理布局和新建义务教育学校，保障农村偏远地区孩子方便就学。聚焦&ldquo;择校热&rdquo;&ldquo;大班额&rdquo;问题，积极扩增义务教育学位，消除大班额500个。聚焦学前教育&ldquo;入园难、入园贵&rdquo;的问题，大力发展公办园，积极扶持民办园，加强小区幼儿园配建及移交工作，购买普惠性民办幼儿园园位8万个。聚焦中小学生学业负担过重的问题，规范校外培训行为，清理整顿无证无照机构。大力实施&ldquo;普通高中改造计划&rdquo;，扩大高中阶段教育资源供给。支持铁道职院入园发展，完成市属三所职业院校的基建扫尾工作，创建全国产教融合示范市，打造全国有重要影响的一流职业教育基地。加强教师队伍建设，完善农村教师公费定向培养机制，努力提高教师政治地位、社会地位和职业地位，让人民教师有更好待遇、有更多尊严！

加快健康株洲建设。深化医药卫生体制改革，推进现代医院管理制度试点，着力解决看病难、看病贵等问题。提高基本医保和大病保险保障水平，扩大用药保障范围。加强全科医生队伍建设，推进分级诊疗。完成市中心血站业务用房改扩建工程，加快市三三一医院门急诊综合大楼等项目建设。坚持中西医并重，传承发展中医药事业。支持社会办医，推进医养结合，巩固国家居家和社区养老服务改革试点成果。完成适龄妇女&ldquo;两癌&rdquo;免费检查7.8万人、孕产妇免费产前筛查2.5万人。

繁荣文化体育事业。加快公共文化服务标准化均等化建设，确保市博物馆、美术馆搬迁工程竣工开馆。实现乡镇综合文化站和村级综合文化服务中心全覆盖。积极推广&ldquo;门前三小&rdquo;模式。办好中国&middot;株洲首届国际女子羽毛球团体锦标赛、第二届中国&middot;株洲国际赛车文化节、第六届株洲市全民健身节、湘江城市马拉松赛等体育赛事。深入推进全民阅读工作，办好第十二届株洲读书月活动，努力将株洲打造成书香之城。

加强社会治理。大力开展&ldquo;城乡统筹&middot;幸福株洲&rdquo;创新社会治理系列行动，加强城乡社区治理和乡镇政府服务能力建设。落实重大安全隐患治理&ldquo;一单四制&rdquo;，持续开展&ldquo;落实企业安全生产主体责任年&rdquo;活动，坚决防范和遏制重特大事故发生。不断深化平安株洲创建，持续开展信访工作&ldquo;三无&rdquo;创建活动，深入推进扫黑除恶专项斗争和新一轮三年禁毒人民斗争。全面推进&ldquo;雪亮工程&rdquo;建设，新建摄像头6000个，完善社会治安立体化防控体系。深入开展国家食品安全示范城市创建，推进小作坊、小餐饮、小食品店提质改造工程，坚决把好人民群众饮食用药安全的每一道关口。同时，扎实做好国防教育、国防动员、双拥共建、人民防空等工作，大力支持驻株部队改革发展，巩固军政军民团结。

各位代表！伟大事业不是等得来、喊得来的，而是拼出来、干出来的。新的一年，我们必须以奋斗精神、奋发姿态，积极担当，不断加强政府自身建设，全力推动各项事业全面进步。务必忠诚坚定强担当。树牢&ldquo;四个意识&rdquo;，坚定&ldquo;四个自信&rdquo;，坚决做到&ldquo;两个维护&rdquo;，以信仰、信念、信心铸就强大精神力量，在攻坚克难中诠释忠诚，在推动发展中体现担当。务必依法行政善担当。自觉运用法治思维破解难题、推动发展。健全依法决策机制，重大决策部署实施前，依法及时向人大及其常委会报告情况。依法接受人大的法律监督和工作监督，自觉接受政协的民主监督，主动接受群众监督和舆论监督，认真研究办理人大代表建议、意见和政协提案。积极推行法律顾问和&ldquo;两公&rdquo;律师制度，深入实施&ldquo;七五&rdquo;普法规划，不断提高依法行政水平。务必勇于创新敢担当。冲破思想观念的束缚，突破利益固化的藩篱，鼓起迎难而上的勇气，保持敢为人先的锐气，提振开拓进取的志气，积极应变、主动求变，在新时代改革开放中开创新气象、实现新作为。务必改进作风勤担当。深入推进政府系统全面从严治党工作，深入开展&ldquo;马上就办、真抓实干&rdquo;活动，驰而不息改作风、纠&ldquo;四风&rdquo;。做好中央、省委巡视反馈问题意见整改，强化巡视巡察、审计和督查结果运用，扎牢织密制度笼子，坚决惩治腐败行为。紧紧依靠人民，用脚步丈量民情、用服务增添民利，以干事创业的积极成效取信于民、造福于民。坚持&ldquo;三个区分开来&rdquo;，旗帜鲜明地为敢于担当作为的干部撑腰鼓劲。

各位代表！梦想辽阔，山河春满。让我们高举习近平新时代中国特色社会主义思想伟大旗帜，在省委、省政府和市委的坚强领导下，与时代同行，与人民同心，脚踏实地，真抓实干，为加快建成&ldquo;一谷三区&rdquo;、加快实现基本现代化不懈奋斗！
